# Ciprofloxacin Loaded Nanostructured Lipid Carriers Incorporated into In-Situ Gels to Improve Management of Bacterial Endophthalmitis

**DOI:** 10.3390/pharmaceutics12060572

**Published:** 2020-06-19

**Authors:** Ahmed Youssef, Narendar Dudhipala, Soumyajit Majumdar

**Affiliations:** 1 Department of Pharmaceutical Technology, Faculty of Pharmacy, Kafrelsheikh University, Kafrelsheikh 33516, Egypt; aayousse@go.olemiss.edu; 2 Department of Pharmaceutics and Drug Delivery, School of Pharmacy, University of Mississippi, Oxford, MS 38677, USA; ndudhipa@olemiss.edu; 3 Research Institute of Pharmaceutical Sciences, University of Mississippi, Oxford, MS 38677, USA

**Keywords:** bacterial endophthalmitis, Ciprofloxacin, nanostructured lipid carriers, in situ gel, stability, transcorneal permeability

## Abstract

Bacterial endophthalmitis (BE) is a potentially sight-threatening inflammatory reaction of the intraocular fluids or tissues caused by bacteria. Ciprofloxacin (CIP) eye drops are prescribed as first-line therapy in BE. However, frequent administration is necessary due to precorneal loss and poor ocular bioavailability. The objective of the current research was to prepare CIP containing nanostructured lipid carriers (CIP-NLCs) loaded an in situ gel system (CIP-NLC-IG) for topical ocular administration for enhanced and sustained antibacterial activity in BE treatment. CIP-NLCs were prepared by the hot homogenization method and optimized based on physicochemical characteristics and physical stability. The optimized CIP-NLC formulation was converted into CIP-NLC-IG with the addition of gellan gum as a gelling agent. Furthermore, optimized CIP-NLC and CIP-NLC-IG were evaluated for in vitro release and ex vivo transcorneal permeation studies, using commercial CIP ophthalmic solution (CIP-C) as the control. The optimized CIP-NLC formulation showed particle size, polydispersity index, zeta potential, assay and entrapment efficiency of 193.1 ± 5.1 nm, 0.43 ± 0.01, −32.5 ± 1.5 mV, 99.5 ± 5.5 and 96.3 ± 2.5%, respectively. CIP-NLC-IG with 0.2% *w*/*v* gellan gum showed optimal viscoelastic characteristics. The in vitro release studies demonstrated sustained release of CIP from CIP-NLC and CIP-NLC-IG formulations over a 24 h period. Transcorneal flux and permeability increased 4 and 3.5-fold, and 2.2 and 1.9-fold from CIP-NLC and CIP-NLC-IG formulations, respectively, when compared to CIP-C. The results demonstrate that CIP-NLC-IG could be considered as an alternate delivery system to prolong the residence time on the ocular surface after topical administration. Thus, the current CIP ophthalmic formulations may exhibit improved ocular bioavailability and prolonged antibacterial activity, which may improve therapeutic outcomes in the treatment of BE.

## 1. Introduction

The eye is a pharmacokinetically isolated organ with unique anatomy and physiology. Bacterial endophthalmitis (BE) is a devastating ocular infection that may result in irreversible blindness in the affected eye [1,2]. BE can be a serious complication of intraocular surgery, ocular trauma, and intravitreal injections [3,4]. Most of the reported cases of BE are exogenous [1,5]. Endogenous endophthalmitis results from seeding from blood to the eye by bacteria or fungi, but bacteremia or fungemia may be transient and patients may manifest no symptoms of systemic infection [1,5]. There are more than 1.7 million cataract surgeries performed every year in the United States [6]. It has been reported that treating infectious endophthalmitis on an outpatient basis would lead to an estimated $1.5 to $7.8 million reduction in reimbursements per year [7]. BE is a medical emergency, and diagnosis and treatment are essential for protecting vision [1,5]. Intravitreal antibiotics including vancomycin and a third-generation cephalosporin or aminoglycoside are used to treat BE, while vitrectomy is needed in severe cases [2,3]. Systemic antibiotics alone have little benefit in treating BE, and their value as adjunctive therapy (in addition to intravitreal antibiotics and vitrectomy) in postoperative and other types of exogenous bacterial endophthalmitis is not clear [8,9]. Because the causal organism of BE may be the patient’s normal conjunctival bacterial flora, prophylactic measures aim to reduce the number of ocular surface bacteria before and after any surgery [10,11].

Ocular antibiotics and anti-inflammatory agents can be used before, during, and after ocular surgery for prophylaxis against BE, together with intracameral cefuroxime at the end of surgery [11,12]. Fluoroquinolones are routinely administered topically along with an antiseptic like povidone-iodine before cataract removal [12,13]. Fluoroquinolones remain the most frequently topically administered class of antibiotics in the treatment of ocular infections and BE [2,14]. Ciprofloxacin (CIP) is a broad-spectrum fluoroquinolone bactericidal antibiotic, active against aerobic Gram-positive and Gram-negative bacteria. It is thus effective in the treatment of a wide variety of ocular bacterial infections [14,15]. It is prescribed in the treatment of corneal keratitis [16], endophthalmitis [17], bacterial and allergic conjunctivitis [18], and for many other bacterial infections of the eye [19]. CIP was chosen based on earlier studies addressing its sensitivity profile against bacterial isolates encountered in BE. Vincent et al. performed a sensitivity study for 14 Gram-positive organisms isolated from patients with acute endophthalmitis following cataract surgery during examination in a referral vitreoretinal practice for two years, and sensitivities to moxifloxacin (38%), gatifloxacin (38%), levofloxacin (46%), ofloxacin (44%), and CIP (50%) were reported [20]. Furthermore, Benz et al. reported that 92% of Gram-negative isolates from the vitreous cavity of patients with BE were susceptible to CIP [21].

Topical ocular administration is the most widely adopted, non-invasive route of drug administration to treat ocular diseases because of its ease of administration and patient compliance [22]. The penetration of therapeutic agents to the intraocular sites-of-action is needed to achieve optimal therapeutic outcomes. However, static and dynamic barriers, reflex blinking, tear turnover and nasolachrymal drainage are anatomical and physiological barriers that constrain ocular delivery [22]. Novel drug delivery and nanotechnology-based drug delivery systems to improve penetration into the anterior and posterior segments of the eye have been extensively investigated [19,23,24,25,26,27,28,29].

Nano formulations are easy to formulate, biocompatible, nonirritant, sustain drug release, and enhance ocular bioavailability of therapeutic moieties [22,30]. Nanostructured lipid carriers (NLCs) consist of a blend of liquid and solid lipids which results in a partially crystallized lipid system for drug entrapment. NLCs and solid lipid nanoparticles (SLNs) share similar advantages in terms of protection from various degradation mechanisms, ease of scaling up using high-pressure homogenization technique, biocompatibility, and biodegradability [31,32]. NLCs, however, have been developed as second-generation lipid-based nanoparticles to overcome problems encountered with SLNs, such as low entrapment efficiency (EE) and drug expulsion during storage [19,32].

In-situ gelling (IG) systems have become promising amongst ocular drug delivery platforms because of their ability to reduce the frequency of topical administration. Their advantages are based on a solution to gel phase transition following instillation in the conjunctival cul de sac, triggered by stimuli such as temperature, ionic strength, and pH [33,34]. This leads to prolonged ocular surface residence which may enhance the ocular bioavailability of drugs and therefore lead to better therapeutic outcomes [29,35,36,37]. Moreover, reduction of the pre-corneal elimination of the gel minimizes absorption into systemic circulation during ocular administration and thus may reduce side effects [25].

CIP is commercially available as ophthalmic solutions and ointments (0.3% *w*/*v* CIP base) for ocular administration. Commercial CIP ophthalmic solution requires frequent administration due to poor ocular bioavailability as against blurred vision, itching, redness, eye discomfort, and dryness due to ointment application [22]. With the solubility of CIP being low at tear fluid pH, precipitation takes place after administration of the commercial ophthalmic solutions, leading to low ocular bioavailability of CIP [19,35]. Surface modification of CIP-loaded NLC (CIP-NLC) with polyethylene glycol (CIP-PEG-NLC) was adopted to improve ocular retention and intraocular penetration of CIP. PEG grafted phospholipids with PEG molecular weights between 2 k and 5 k showed enhanced ocular permeation of CIP and delivered the drug to the back of the eye and also increased autoclave stability of CIP-NLC [19]. However, Akash et al. reported that natamycin-loaded PEG-NLCs showed enhanced in vitro and in vivo corneal permeation, and converting NLCs into a gelling system improved the corneal retention [36]. Moreover the entrapment efficiency of CIP in CIP-PEG-NLC formulation was less than 90% [19]. Hence, an alternative delivery system with enhanced corneal residence time and mucoadhesive characteristics could further improve the characteristics of the CIP-NLC formulations.

The objective of the present research was to overcome the potential drawbacks of the eye drops by developing CIP-NLCs and incorporation of ion-sensitive in situ gelling agents (CIP-NLC-IG) that might enhance ocular retention and bioavailability and reduce the frequency of administration. Accordingly, CIP-NLCs were prepared and optimized based on physicochemical characteristics and stability. The optimized CIP-NLC’s were then converted into CIP-NLC-IG with the addition of gellan gum as the gelling agent and further optimized. The optimized CIP-NLCs and CIP-NLC-IG formulations were evaluated for in vitro release and ex vivo permeation and compared against the commercial CIP ophthalmic solution USP 0.3% as a base.

## 2. Materials and Methods

### 2.1. Materials

Ciprofloxacin was purchased from Sigma-Aldrich (St. Louis, MO, USA). Precirol^®^ ATO 5 (Glyceryl distearate) was a generous gift sample from Gattefossé (Paramus, NJ, USA). Amicon^®^ Ultra centrifugal filter devices with regenerated cellulose membrane (molecular weight cut off 100 kDa), Tween^®^ 80, oleic acid, and Poloxamer 188 were acquired from Fischer Scientific (Hampton, NH, USA). Gellan gum was purchased from MP Biomedicals, LLC (Santa Ana, CA, USA). Other chemicals and glassware required for this research like HPLC grade solvents, scintillation vials, centrifuge tubes, HPLC vials were obtained from Fischer Scientific (Hampton, NH, USA). Whole eyes of male albino New Zealand rabbits were obtained from Pel-Freez Biologicals (Rogers, AR, USA).

### 2.2. Methods

#### 2.2.1. Quantification of CIP by HPLC

CIP was quantified using an earlier published HPLC method with some modification and subsequent validation [38]. The HPLC system consisted of a Waters 717 plus auto-sampler coupled with a Waters 2487 Dual λ Absorbance UV detector, a Waters 600 controller pump, and an Agilent 3395 Integrator. The mobile phase consisted of a mixture of phosphate buffer (25 mM, pH 2.4) and acetonitrile (70:30% *v*/*v*) with a flow rate of 1 mL/min. A C_18_ Phenomenex Luna^®^ (5 μ, 250 × 4.6 mm) column was used. The temperature for the analyses was 25 °C, the injection volume was 20 μL, and the UV detection wavelength was set to 290 nm at Absorbance Units Full Scale (AUFS) 1.00. The method was found to be linear over the concentration range of 1–30 μg/mL. The modified method was found to be precise and accurate with a limit of detection (LOD) and limit of quantitation (LOQ) of 0.4 and 1.2 µg/mL, respectively.

#### 2.2.2. Screening of Lipid Excipients

NLCs are developed using solid and liquid lipids as the main components. Hence, many solid and liquid lipids were screened to evaluate the solubility of CIP, for the preparation of the CIP-NLCs. Solid lipids (Compritol^®^ 888 ATO, Dynasan^TM^ 114, Geleol^TM^, Precirol^®^ ATO 5, Dynasan^TM^ 116, Gelucire^TM^ 44/14, Gelucire^TM^ 43/01, Gelucire^TM^ 50/13, Softisan 154) and liquid lipids (castor oil, olive oil, soybean oil, sesame oil, Maisine^®^ CC, oleic acid, Miglyol^®^ 829, Captex^®^ 355 EP and Capryol 90^TM^) were screened. Briefly, about 10 mg of CIP, accurately weighed, was added to 100 mg of the melted lipid at 80 ± 2 °C under continuous magnetic stirring at 2000 rpm for 10 mins in 3-mL-capacity glass vials. The CIP lipid mixtures were then allowed to cool. All the CIP lipid mixtures were then visually observed for the precipitation of CIP.

#### 2.2.3. Preparation of CIP-NLCs

CIP-NLCs were prepared by homogenization method [19]. An aqueous phase was prepared using surfactants (Tween^®^ 80 and poloxamer 188) and glycerin in double distilled water, and heated to 80 ± 2 °C. The lipid phase consisted of CIP (0.1% *w*/*v*) dissolved in Precirol^®^ ATO 5 and oleic acid mixture and heated at a temperature of 80 ± 2 °C. Then, the hot aqueous phase was added to the molten lipid phase drop by drop under continuous magnetic stirring at 2000 rpm for10 mins to form a homogeneous mixture. The coarse emulsion was then further emulsified at 15,000 rpm for 5 mins using a T25 digital Ultra-Turrax (IKA, Germany), to form a hot emulsion. Cooling this emulsion to room temperature led to the solidification of the solid lipids and formation of the NLCs.

#### 2.2.4. Preparation of CIP-NLC-IG

CIP-NLC-IG formulations were prepared as per the CIP-NLC method. However, the water used in the preparation of NLCs was divided into two parts, one part was used to dissolve the gellan gum (0.2–0.4% *w*/*v*) and the other for the preparation of the aqueous phase as mentioned above. Both parts were heated to 80 ± 2 °C and then transferred simultaneously to the hot lipid–drug solution, followed by emulsification and homogenization, as described for the CIP-NLCs, to form in situ gels.

#### 2.2.5. Control Formulation

Commercial CIP ophthalmic solution USP 0.3% as a base (Alcon Laboratories, Texas, USA; Lot # 295240F) was diluted using isotonic phosphate-buffered saline (IPBS) and Dulbecco’s phosphate-buffered saline (DPBS) to 0.1% *w*/*v* (CIP-C) for in vitro release and transcorneal permeation studies, respectively.

### 2.3. Characterization of CIP-NLCs

#### 2.3.1. Measurement of Particle Size (PS), Polydispersity index (PDI) and Zeta Potential (ZP)

PS, ZP, and PDI of the CIP-NLCs prepared were measured by photon correlation spectroscopy using a Zetasizer Nano ZS Zen3600 (Malvern Instruments, MA, USA) at 25 °C in disposable folded clear capillary cells. Particle size and PDI measurements were attained using a helium-neon laser, and the data were analyzed based on the volume distribution. The samples were diluted 100 times with filtered bi-distilled water and measured in triplicate.

#### 2.3.2. Assay (CIP Content)

CIP content in the CIP-NLCs was determined using the lipid precipitation method wherein an accurately measured volume of the NLCs (10 μL) was extracted in a 1:1 binary mixture of 0.1N HCl and 200 proof alcohol (990 μL). The mixture was centrifuged (AccuSpin 17R centrifuge, Fisher Scientific, Waltham, MA, USA) at 13,000 rpm for 20 mins, and the supernatant was analyzed for CIP content, using the HPLC method described above, following appropriate dilution. The CIP content (assay) was used to calculate the percentage entrapment efficiency (%) of CIP in the CIP-NLCs.

#### 2.3.3. Entrapment Efficiency (EE)

The EE (%) of CIP in CIP-NLCs was determined by measuring the amount of free drug in the aqueous phase of the original formulations. The EE was determined by ultrafiltration with a 100 kDa centrifugal filter device (Amicon Ultra, Hanover, IL, Fisher Scientific, USA). An aliquot (400 μL) of the formulation was added to the sample reservoir and centrifuged at 13,000 rpm for 20 mins. The filtrate was analyzed for drug content following proper dilution. All measurements were carried out in triplicates, and the % EE was calculated by using the following equation:

(1) $$\%\;\mathrm{EE}=\left[\frac{\mathrm{amount}\;\mathrm{of}\;\mathrm{CIP}\;\mathrm{determined}\;\mathrm{in}\;\mathrm{assay}-\mathrm{amount}\;\mathrm{of}\;\mathrm{unentrapped}\;\mathrm{CIP}}{\mathrm{amount}\;\mathrm{of}\;\mathrm{CIP}\;\mathrm{weighed}}\right]\times 100$$

#### 2.3.4. Stability Studies of CIP-NLCs

Stability of the optimized CIP-NLC formulations was studied at refrigerated and room temperature conditions [39]. Briefly, a volume of 5 mL of NLCs dispersion was filled into 20 mL scintillation glass vials and stored under refrigerated (4 ± 2 °C) and room temperature (25 ± 2 °C) conditions. The formulation was evaluated for analyzing the changes in PS, PDI, ZP, % drug content, and EE upon storage at predetermined time intervals up to three months.

#### 2.3.5. Fourier Transform Infrared Spectroscopy (FTIR)

The interaction of CIP with other formulation components was analyzed using FTIR spectroscopy. The studies were performed on an Agilent Technologies Cary 660 (Santa Clara, CA, USA) instrument. The pure drug, pure lipid excipients along with their physical mixtures and the final blank and drug-loaded formulation were studied for any interactions. The bench was equipped with an ATR (Pike Technologies MIRacle ATR, Madison, WI, USA) that was fitted with a single-bounce, diamond-coated ZnSe internal reflection element. The scanning range was 800–4000 cm^−1^.

### 2.4. Characterization of CIP-NLC-IG

#### 2.4.1. Drug Content

CIP content in the CIP-NLC-IG formulation was determined similarly to CIP-NLCs wherein an accurately measured volume of the IG formulation (10 μL) was extracted in a 1:1 binary mixture of 0.1N HCl and 200 proof alcohol (990 μL). Then, the mixture was centrifuged (AccuSpin 17R centrifuge, Fisher Scientific, USA) at 13,000 rpm for 20 mins and the supernatant was analyzed for drug content using HPLC following dilution.

#### 2.4.2. Rheological and In Vitro Gelling Characteristics of CIP-NLC-IG

Simulated tear fluid (STF) with a pH of 7 ± 0.2 was prepared by adding 0.0084% Calcium Chloride, 0.678% Sodium Chloride, 0.138% Potassium Chloride, and 0.218% Sodium bicarbonate in deionized water was used as a medium for studying gelling behaviour [37]. The gelling time (GT) is the time required for CIP-NLC-IG formulations to form a gel upon addition to STF without agitation. Gel residence time (GRT) was the time required for the gel to dissolve or break based on visual inspection every hour for the first 8 h and then every 4 h for the rest of the experiment. The GT and GRT of the formulations with different concentration of gellan gum were determined by dropping 50 µL of the CIP-NLC-IG in 2 mL of freshly prepared STF and maintaining the temperature of the glass vial at 34 °C in a shaking water bath (Precision^TM^, Fisher Scientific, USA) set at 100 rpm for 24 h.

The viscosity of CIP-NLC-IG was measured using a Brookfield cone and plate viscometer (LV-DV-II+ Pro Viscometer, Middleboro, MA, USA). Briefly, 0.5 mL of each formulation was placed in the cup plate after adjusting the gap between the cone and plate. The samples in the cup were maintained at 25 °C using a circulating water bath. A CPE 52 spindle was operated at 2 rpm and the viscosity was recorded from Rheocalc software. To determine the viscosity of the hydrogels, 0.5 mL of a mixture of CIP-NLC-IG formulation without and with STF (50:7) was placed in a cup plate, and a similar procedure was repeated to record viscosity (*n* = 3).

#### 2.4.3. Stability Studies of CIP-NLC-IG

Optimized CIP-NLC-IG formulation was observed for changes in GT, GRT, viscosity, and % drug content upon storage at 4 ± 2 °C and 25 ± 2 °C for one month.

#### 2.4.4. In Vitro Release Studies of CIP-NLC and CIP-NLC-IG

Based on the earlier reported solubility studies of CIP in different dissolution media [19], IPBS (pH 7.4) containing 2.5% *w*/*v* randomly methylated beta-cyclodextrin (RMβCD) was used as the receiver medium for in vitro release and transcorneal permeation studies. In vitro release of CIP from the respective formulations, such as CIP-C, CIP-NLCs, and CIP-NLC-IG, were evaluated using the diffusion method. Two hundred microliter of the formulations were added into a 0.5 mL cup-like design Thermo Scientific™ Slide-A-Lyzer™ MINI Dialysis Device (10 K molecular weight cutoff) which was considered as the donor compartment and then mounted on the top of a 20 mL scintillation vial as the receiver compartment. STF was added to CIP-NLC-IG formulation at a ratio of 50:7 to form a gel inside the MINI Dialysis Device. The content of the receiver compartment was maintained under continuous magnetic stirring at 34 °C. Aliquots (1 mL) were withdrawn from the receptor vials and replaced with an equal volume of the 2.5% *w*/*v* RMβCD in IPBS (pH 7.4) solution at nine-time points (0.5, 1, 2, 3, 4, 6, 8, 12, and 24 h). Donor CIP concentrations were maintained at 0.1% *w*/*v* for all the formulations. Samples were quantified using HPLC. The CIP release data obtained were analyzed for release kinetics using Microsoft (MS) Office excel statistical functions (Office365, 2016, USA), according to different mathematical models to figure out the possible release mechanism. Linear regression equations were used, and goodness of fit was evaluated based on the coefficient of determination (R^2^) [40].

#### 2.4.5. Ex Vivo Transcorneal Permeation Studies

Ex vivo transcorneal permeation of CIP from CIP-NLCs and CIP-NLC-IG formulations, in comparison with CIP-C, were studied on corneas excised from rabbit whole eyes, shipped from Pel-Freez Biologicals (Rogers, AR, USA). The eyes were stored in Hanks’ balanced salt solution under cold conditions and shipped overnight. The corneas were carefully excised and used for the permeation studies immediately upon their arrival. The isolated corneas were washed in IPBS solution, pH 7.4. The cornea was clamped in between the two half-cells (Valia-Chien cells (PermeGear, Inc1)) with the epithelial surface facing the donor cell which contains the formulations. Optimized CIP-NLCs, CIP-NLC-IG, and CIP-C were used as donor formulations. The receiver compartment consisted of 2.5% *w*/*v* RMβCD in IPBS solution with pH 7.4 for all the permeation studies. The contents of the receiver compartment were under continuous magnetic stirring and maintained at 34 °C with the help of a circulating water bath. Aliquots were withdrawn from the receiver chamber at different time points (15, 30, 60, 90, 120, and 180 mins) and replaced with an equal volume of 2.5% *w*/*v* RMBCD in IPBS. The samples were analyzed using the HPLC method described above. Analysis for all the samples was carried out in triplicate. The cumulative amount of drug permeated (M_n_), steady-state flux (Jss), and transcorneal permeability (P_eff_), across the rabbit cornea, were estimated to study the transport of CIP across rabbit cornea. The cumulative amount of CIP was calculated using the following equation:

(2) $$M_{n}=V_{r}C_{r\left(n\right)}+\overset{x=n}{\underset{x=1}{\sum }}V_{s\left(x-1\right)}C_{r\left(x-1\right)}$$

where, ***n*** is sampling time point; ***V_r_*** and ***V_S_*** are the volume in the receiver chamber (mL) and the volume of the sample collected at the nth time point (mL), respectively and ***C_r(n)_*** is the concentration of the drug in the receiver chamber medium at the nth time point (µg/mL).

The rate of CIP transport across rabbit cornea was calculated using the slope of the cumulative amount of CIP transported versus time plot. The steady-state flux of CIP was determined using the following equation:

(3) Jss=(dM/dt)/A

where, ***M*** is the cumulative amount of drug transported and ***A*** is the surface area of the cornea (0.636 cm^2^).

The transcorneal permeability of CIP was calculated by the following equation:

(4) $$P_{\mathrm{eff}}=\frac{Jss}{Donor\;concentration}$$

#### 2.4.6. Statistical Analysis

A one-way analysis of variance (ANOVA) (IBM SPSS Statistics software, SPSS 25, USA) was used to analyze the data, and the statistically significant difference between the set of formulations was observed at a ‘*p*’ value less than 0.05 (*p* < 0.05).

## 3. Results and Discussion

### 3.1. Screening of Lipid Excipients

A selection of suitable lipids is essential for the successful formulation of NLCs for poorly soluble drugs like CIP, as it directly affects drug loading and the EE of NLCs [41]. Better EE can also be achieved by adding liquid lipids which form incomplete lattices and load more drug [42,43]. The lipids which did not demonstrate any drug precipitation at the bottom of the glass vials after melting and cooling of the CIP-lipid mix were selected as presented in Table 1. Based on the results, Precirol^®^ ATO 5 and oleic acid were chosen as solid and liquid lipids, respectively, for the preparation of CIP-NLCs.

### 3.2. Physical Characterization of CIP-NLC

The main aim of the present work was to develop NLCs and their corresponding IG to enhance penetration and provide a controlled-release, ophthalmic dosage form for CIP. The influence of different surfactant concentrations, total lipid to surfactant ratio, and different homogenization speeds on the PS and PDI, and ZP was studied. Precirol^®^ ATO 5 and oleic acid were used as solid lipid and liquid lipids, respectively, and Tween^®^ 80 and Poloxamer 188 were used as surfactants in the CIP-NLC development. The detailed composition of the final optimized formulations is presented in Table 2. In general, a solid to liquid lipid ratio of 3:1 resulted in the smaller PS of NLC formulations [40,44].

*Effect of Tween^®^ 80 concentration:* Tween^®^ 80 is the primary surfactant in the formulation of CIP-NLCs. Therefore, the effect of different Tween^®^ 80 concentrations was studied at 15,000 rpm homogenization speed and results are shown in Table 3. Increasing Tween^®^ 80 concentrations from 0.75 to 2% *w*/*v* in NLC placebos decreased PS significantly, from 384.4 ± 7.9 to 142.3 ± 3.9 nm.

*Effect of homogenization speed:* The effect of homogenization speed on placebo NLC formulations developed with 2% *w*/*v* Tween^®^ 80 was studied. PS and PDI decreased from 314.5 ± 8.1 to 142.3 ± 3.9 nm and from 0.49 ± 0.05 to 0.38 ± 0.01, respectively, with increasing homogenization speeds (14,000 to 15,000). However, there was no significant change in ZP. In addition, increasing homogenization speed to more than 15,000 rpm resulted in blackening in the bulk of the formulation and also precipitation. Hence, 15,000 rpm was chosen as the optimum homogenization speed for NLCs preparation.

*Effect of total lipids to surfactant ratio:* The effect of increasing total lipid to surfactant ratio during NLC placebo preparation is shown in Table 4. Increasing lipid to surfactant ratio (2:1 to 3:1) increased PS and PDI from 142.3 ± 3.9 to 226.0 ± 11.2 nm, and 0.38 ± 0.01 to 0.60 ± 0.05, respectively. However, there was no significant change in ZP. Therefore, a 2:1 lipid to surfactant ratio was selected for the preparation of drug-loaded NLCs.

Thus, increasing surfactant concentrations had a significant effect on PS which could be attributed to a substantial reduction in surface tension and surface-free energy during homogenization due to high shear conditions [45]. The increase in homogenization speed from 14,000 to 15,000 rpm resulted in a simultaneous increase in breaking energy, resulting in NLC formulation with smaller PS and thus, a narrow PDI [46]. A homogenization speed above 15,000 resulted in the formulation being blackened, which may be attributed to the charring of lipids attributed to very high shear energy. On increasing the amount of lipid to surfactant ratio (2:1 to 3:1), the NLC dispersion becomes more viscous and the applied shear may be not enough for size reduction, therefore, PS and PDI tend to increase [47,48].

**Figure 1:**
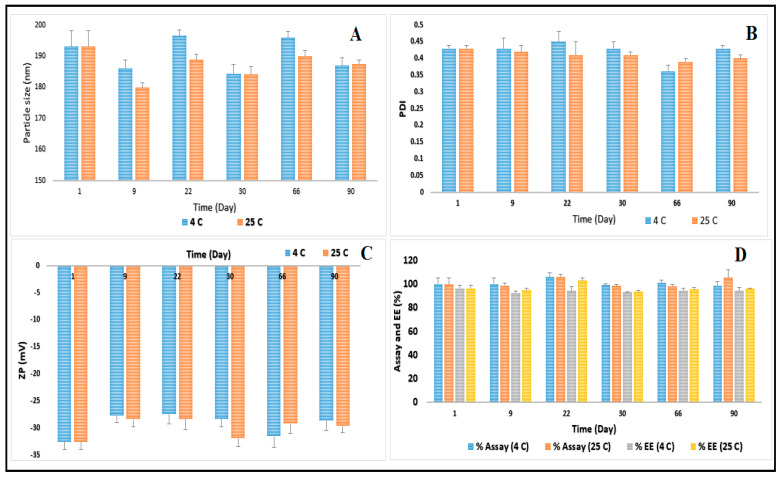
(**A**) particle size, (**B**) polydispersity index, (**C**) zeta potential, and (**D**) assay and entrapment efficiency of optimized CIP-NLC formulation over three months storage at 4 ± 2 °C and 25 ± 2 °C (mean ± SD, *n* = 3).

The size of the nanoparticles is an important factor for adhesion to and interaction with the target cells. Particles of 100–200 nm size can best be internalized through receptor-mediate endocytosis, while larger particles need to be absorbed through phagocytosis [49,50,51]. The optimized CIP-NLC formulation showed PS, PDI, and ZP of 193.1 ± 5.1 nm, 0.43 ± 0.01, and −32.5 ± 1.5 mV, respectively, as depicted in Figure 1. Sai et al. reported that CIP-PEG-NLC (optimized formulation) formulation showed PS, PDI, and ZP of 180.6 ± 13.0 nm, 0.31 ± 0.01, and −1.8 ± 0.08 mV, respectively, and the significant difference in ZP was due to the surface charge neutralization by PEG [19]. Furthermore, Sai et al. used homogenization coupled with the probe sonication method for the preparation of CIP-PEG-NLC in two successive steps. However, in the current study, the homogenization method was only sufficient for the development of CIP-NLC formulation in one single step. Additionally, only one solid lipid was used in the preparation of CIP-NLCs formulation compared to two different solid lipids in CIP-PEG-NLC formulation which could be a cost-effective approach [19]. The magnitude of ZP is an indication of the stability of colloidal dispersion. ZP of −20 to −30 mV is required for electrostatic stabilization for dispersed systems [52]. Steric stabilizers such as Poloxamer 188, a non-ionic surfactant, can be added to the nano lipid dispersions, which promotes the electrostatic stabilization because it can reduce the electrostatic repulsion between the particles by establishing a coat around the surface for keeping the stability of lipid nanoparticles [53].

### 3.3. Assay and EE for Optimized CIP-NLC

The type of liquid and solid lipids and surfactant plays a critical role in drug encapsulation into NLCs. The drug needs to be dissolved or dispersed in the oily phase before homogenization to achieve the maximum drug entrapment and minimize drug precipitation after the preparation of the lipid-based system [54]. Higher EE in the NLCs is obtained due to the blending of solid and liquid lipids, creating a disordered structure that provides higher space for drug loading. Moreover, the drug is more soluble in liquid lipids than solid lipids [41,55].

Drug content and EE of the optimized CIP-NLC formulation was 99.5 ± 5.5 and 96.3 ± 2.5%**,** respectively. The lower the EE within the lipid matrix, the more the drug in the aqueous phase which could lead to the precipitation of the free drug at the ocular surface due to low solubility at tear fluid pH. In earlier studies, Sai et al. reported that CIP-PEG-NLC formulations showed EE of 83.6 ± 4.7%. However, Sharma et al. reported an EE close to 85% when the CIP was loaded in SLNs [56]. The EE of CIP-NLC formulation was significantly higher than earlier reported studies. Higher EE could be explained by increased space due to imperfect lipid matrix structure created between the oleic acid chain and the esters of palmitic (C_16_) and stearic (C_18_) acids of Precirol^®^ ATO 5, permitting more drug to be accommodated.

### 3.4. Stability Studies of CIP-NLC

The physical stability of the optimized CIP-NLC formulation was determined by the storage of the samples at refrigerated and room temperature conditions over 90 days. The effect of the storage condition on PS, PDI, ZP, drug content, and EE is shown in Figure 1. The optimized CIP-NLC formulation did not show any aggregation or cracking upon visual inspection, until 90 days (last time point) at refrigerated and room temperature. PS of the optimized CIP-NLC formulation ranged from 184.4 to 196.6 nm and from 179.8 to 193.1 nm, PDI ranged from 0.36 to 0.45 and from 0.39 to 0.43 and ZP ranged from −27.4 to −32.5 mV and from −28.4 to −32.5 mV, at refrigerated and room temperature, respectively. There was no significant difference (*p* > 0.05) observed in PS, PDI, and ZP after three months of storage at 4 ± 2 °C and 25 ± 2 °C which is attributed to the presence of Tween^®^ 80 surfactant and the steric stabilizer Poloxamer 188. Similarly, no significant changes also noticed in drug content and EE of CIP-NLC over 90 days which could be due to the partially crystallized structure of NLCs.

### 3.5. Characterization of CIP-NLC-IG

CIP-NLC-IG formulations were prepared by varying the concentrations of gellan gum (0.2–0.4% *w*/*v*). The rheological properties and % drug content of CIP-NLC-IG formulation are shown in Table 5. The GT occurred instantaneously and GRT was more than 24 h for all different gellan gum concentrations. The drug content of CIP-NLC-IG2 formulation showed a % drug content of 94.8 ± 2.4%. The viscosity of the CIP-NLC-IG formulations was the selection criterion for the optimum gellan gum concentration for further evaluation. For ophthalmic solutions, the viscosity up to 50 cP is most widely accepted, allowing for easy topical ocular application [57]. Although the viscosity of CIP-NLC-IG3 formulation was within the accepted range in the absence of STF, it showed high viscosity after the addition of STF. The formulation with 0.4% *w*/*v* (CIP-NLC-IG4) of gellan gum was not in the range of ideal ophthalmic solution because, at that concentration of the gelling agent, a highly viscous dispersion formed. The viscosity of the CIP-NLC-IG (0.2% *w*/*v*) was found to be 7.9 ± 0.2 cP and 55 ± 2.7 cP without and with STF, respectively. After the addition of STF, there was an increase in the viscosity due to the cross-linking of the polymer chain by the mono or divalent cations present in the STF [25,29,37].

GT and GRT are the critical properties, which determine the efficiency of the ocular IG systems [25]. GT of <5 s indicated the immediate sol-to-gel phase transition of CIP-NLC-IG in the ocular milieu. The abundance of mono or divalent cations in the tear fluid results in the rapid formation of an intact hydrogel matrix by cross-linking gellan gum to form double helix aggregates [58]. Hence, the viscoelastic and work of adhesion properties of the hydrogels increased linearly with the concentration of the gellan gum in the CIP-NLC-IG formulations suggesting longer precorneal residence [25,58].

### 3.6. Stability Studies of the Optimized CIP-NLC-IG

**Figure 2:**
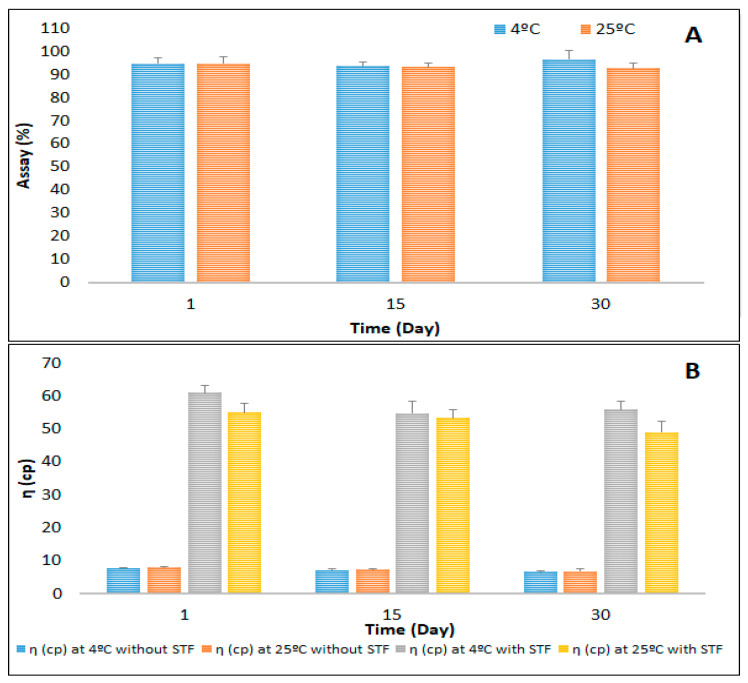
Stability of optimized CIP-NLC-IG formulation stored at 4 ± 2 °C and 25 ± 2 °C over one month—(**A**) drug content and (**B**) viscosity (mean ± SD, *n* = 3).

From the rheological properties, CIP-NLC-IG with 0.2% *w*/*v* concentration showed ideal ophthalmic formulation viscosity, high drug content with instantaneous GT, and prolonged GRT. Hence, this formulation was evaluated for stability. The stability of CIP-NLC-IG2 was determined by storage at 4 ± 2 °C and 25 ± 2 °C and results are shown in Figure 2. The viscosity (η) of the CIP-NLC-IG2 formulation with and without STF, did not show a significant difference during the one-month stability study. CIP-NLC-IG2 were further tested for in vitro release and transcorneal permeation studies.

### 3.7. FTIR Studies

**Figure 3:**
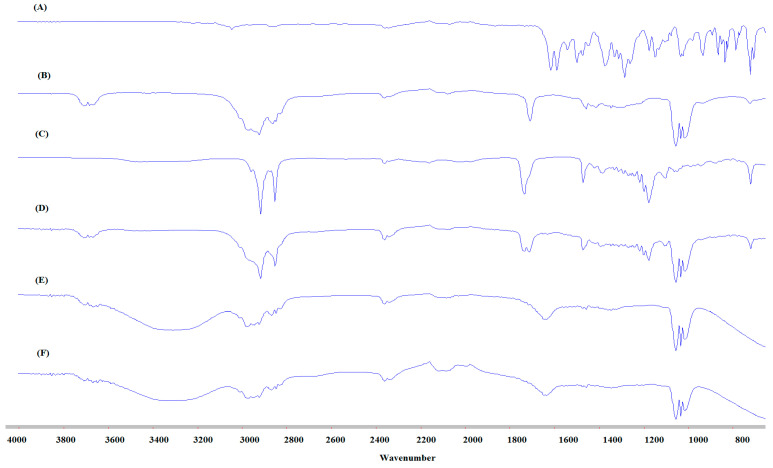
Fourier Transform Infrared Spectroscopy (FTIR) spectra of (**A**) Ciprofloxacin (CIP), (**B**) Oleic acid, (**C**) Precirol^®^ ATO 5, (**D**) physical mixture (CIP, oleic acid and Precirol^®^ ATO 5), (**E**) blank NLC, and (**F**) CIP-NLC formulation.

FTIR spectrums were collected for pure CIP, both solid and liquid lipids, i.e., Precirol^®^ ATO 5, oleic acid, physical mixture (CIP, Precirol^®^ ATO 5 and oleic acid) and blank and drug-loaded NLC formulations to confirm that there was no interaction between the excipient and the drug during the formulation process. The FTIR spectra of CIP, lipids, and NLC formulations are presented in Figure 3. The FTIR spectra of the pure oleic acid present the following characteristic peaks: a wide and intense band between 2880 cm^−1^ and 3006 cm^−1^ due to O-H bond, the band is centered at 2921 cm^−1^; C=O stretching band at 1708 cm^−1^ is due to a dimeric oleic acid, and angular deformation outside the plain of O-H bond gives a band at 1013 cm^−1^ and is characteristic of the dimeric oleic acid [59,60]. The FT-IR spectra of the pure Precirol^®^ ATO 5 present the following characteristic bands: major peak for % transmittance shows at 1730 cm^−1^ due to C=O stretching and 2913 cm^−1^ of C-H stretching, which are characteristic of Precirol^®^ ATO 5 (glyceryl palmitostearate) [61]. For CIP, spectra showed carbonyl stretching at 1722 cm^−1^ and stretching vibration of C-F at 1290 cm^−1^. The band at 3043 cm^−1^ and 2918 cm^−1^ is the C-H stretching vibration of the phenyl ring of CIP [62]. There are no peaks for the drug in the physical mixture FTIR spectrum due to high solubility in both liquid and solid lipids. Blank and drug-loaded NLCs have a similar FTIR spectrum with O-H stretching broad peak at 3200–3600 cm^−1^ observed for water which makes the external phase. The FTIR spectra did not show the characteristic CIP peaks in the blank and CIP-NLC formulations due to the dispersion of the drug within the lipid matrix; the observed peaks are from certain functional groups of the lipid excipients. 

### 3.8. In Vitro Release Studies

**Figure 4:**
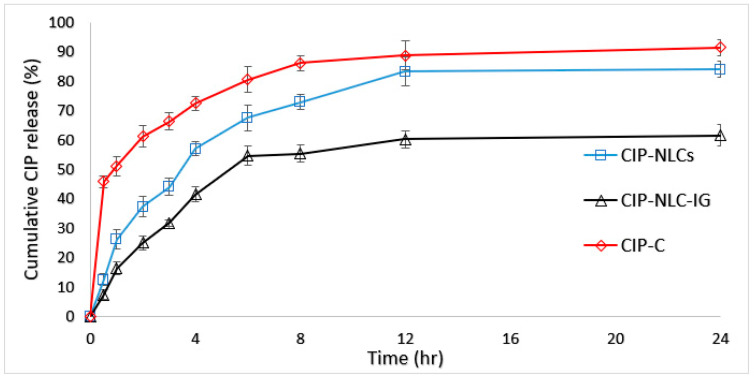
In vitro release of CIP from CIP-NLCs, CIP-NLC-IG2 and CIP ophthalmic solution (CIP-C) through Thermo Scientific™ Slide-A-Lyzer™ MINI Dialysis device (10K MWCO) (mean ± SD, *n* = 3).

The in vitro release of CIP from the optimized CIP-NLC, CIP-NLC-IG2, and CIP-C formulations were studied across Slide-A-Lyzer™ mini Dialysis device (10K MWCO) and results are depicted in Figure 4. The cumulative percentage releases of CIP from CIP-C, CIP-NLC, and CIP-NLC-IG2 were 91.5 ± 1.4%, 82.5 ± 0.1%, and 61.7 ± 0.2%, respectively within the time course of the study (24 h). CIP-C solution showed a cumulative percentage release of 80.7 ± 2.7% after 6 h, and this came in accordance with the cumulative percentage release (83.5%) reported by Sai et al. However, CIP-NLC-IG2 formulation showed a slightly higher percentage cumulative drug release (50.7 ± 0.3) compared to CIP-PEG-NLC (44.5%) formulation after 6 h [19,35]. This is not surprising as the formulation studied by Sai et al. had a three-fold higher drug load. Both CIP-NLC and CIP-NLC-IG2 formulations sustained the release of CIP compared to 0.1% *w*/*v* CIP-C solution.

In order to find the possible release pattern from optimized CIP-NLC, CIP-NLC-IG2, and CIP-C formulations, the release data were analyzed to check the goodness of fit for zero-order release, first-order release, Higuchi [63,64] and Korsmeyer–Peppas model [40,65]. The model that showed the highest R^2^ value was considered as the best model to describe release kinetics. The R^2^ value for each model is presented in Table 6. The highest value of the coefficient of determination (R^2^ = 0.9955) was observed for Korsmeyer–Peppas model, followed by the Higuchi’s (R^2^ = 0.9867), first-order (R^2^ = 0.9849), and zero-order (R^2^ = 0.9301) models for CIP-NLC formulation. However, for CIP-NLC-IG formulation, the highest value of the coefficient of determination (R^2^ = 0.9985) was also observed for Korsmeyer–Peppas model, followed by the first order (R^2^ = 0.9956), Zero-order (R^2^ = 0.9768), and Higuchi (R^2^ = 0.9694) models. Similar release kinetics were reported in earlier studies for lipid nanoparticles [66,67] and hydrogels loaded with lipid nanoparticles [67]. The slope (*n*) of the Korsmeyer–Peppas model for CIP-NLC and CIP-NLC-IG2 formulation was 0.6 and 0.7, respectively which indicated non-Fickian (0.5 < *n* < 1) or anomalous drug release profile controlled by erosion and diffusion mechanisms [68,69].

Sustained drug release from the lipid matrix could be due to drug embedded and entrapped in the solid lipid matrix, which leads to prolonged drug release [70,71]. The reason for sustaining drug release from IG formulation could be due to the solid nature of the lipid matrix and the higher viscosity of the gel, which slows drug diffusion [72].

### 3.9. Ex Vivo Transcorneal Permeation

**Figure 5:**
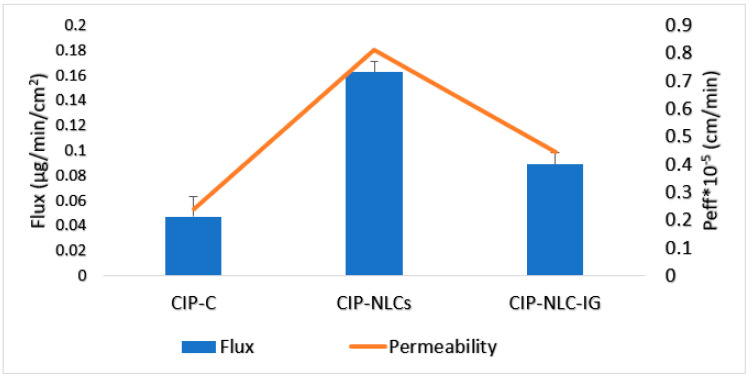
Transcorneal flux and permeability of CIP from optimized CIP-NLC, CIP-NLC-IG2, and CIP-C through the isolated rabbit cornea (mean ± SD, *n* = 3).

About 30 µL of STF was added to the donor compartment along with CIP-NLC-IG2, to form a gel at the corneal surface, to imitate the in vivo conditions. The transcorneal flux and permeability coefficient for CIP-NLCs, CIP-NLC-IG2, and CIP-C are represented in Table 7 and Figure 5. The flux of the CIP from CIP-NLC, CIP-NLC-IG2, and CIP-C formulation was found to be 0.16 ± 0.01 μg/min.cm^2^, 0.09 ± 0.01 μg/min.cm^2^ and 0.04 ± 0.01 μg/min.cm^2^, respectively through rabbit isolated cornea. The transcorneal permeability from CIP-NLC, CIP-NLC-IG2 and CIP-C formulation was 8.1 ± 0.4 × 10^−5^ cm/min, 4.4 ± 0.4 × 10^−5^ cm/min and 2.3 ± 0.8 × 10^−5^ cm/min, respectively. In vitro transcorneal flux of CIP from CIP-NLCs and CIP-NLC-IG2 formulations was 4 and 2.2-fold greater than that achieved with CIP-C formulation. However, the transcorneal permeability of CIP from CIP-NLC and CIP-NLC-IG2 formulations was 3.5 and 1.9-fold greater than that achieved with CIP-C formulation.

The reasons for the enhanced corneal penetration from the optimized CIP-NLC formulation could be due to the following reasons: NLCs are capable of enhancing drug permeation through the cornea because their lipid may interact with the tear film’s oily layer, allowing carriers to stay in the conjunctival sac for a long time, where they act as a drug depot and resist washing away by tear fluid [73,74]. They can also form a film on the corneal epithelium; consequently, the drug is released slowly [73]. NLCs nano-size range can be internalized by the receptor-mediated endocytosis uptake mechanism through the corneal cells [19,51,75]. Gellan gum is an anionic polysaccharide polymer with sol-to-gel transformation characteristics in the presence of cations, and thus, is utilized as an electrolyte sensitive gelling agent. Higher mucoadhesion of polymer with cellular glycoproteins through hydrophobic interactions/van der Waals forces/ionic/hydrogen bonding leads to longer contact time of hydrogel with the biological membrane. Gellan gum also has a thickening property owing to the aggregation of the double helices, resulting from decreased repulsion that induced by cation binding to the helices in specific coordination sites around the carboxylate groups of the polymer, and these cations are present in tear fluid [76].

In earlier studies, Sai et al. reported that transcorneal flux of CIP from the optimized CIP-PEG-NLC formulation was almost three-fold higher than commercial eye drops (0.3% *w*/*v*), but they used 0.3% *w*/*v* drug load. A similar fold increment in the flux (four-fold with NLC and 2.2-fold with IG) was observed with 0.1% *w*/*v* drug load in this study [19]. In future studies, different trials to increase drug load and surface modification with pegylation will be undertaken to further increase drug loading and corneal permeability. Similar outcomes were also observed for NLC-IG loaded with nepafenac prepared by Shihui et al. The transcorneal permeability and flux of nepafenac was enhanced by 1.9 and 2.4-fold, respectively, compared with the marketed eye drops [77]. Karthik et al. reported that the transcorneal flux of natamycin from natamycin transferosomes in situ gel based on gellan gum was six-fold greater than that achieved with control suspension [25]. Furthermore, Akshaya et al. reported a 9.3-fold enhancement in the transcorneal permeability of an ocular in situ gelling system loaded with SLNs of triamcinolone acetonide compared to control formulation [29]. Various nanoparticle-converted IG delivery systems exhibited enhanced permeability and also delivered the drug to the back of the eye [29]. This work demonstrated that NLCs and their in situ gel enhanced the absorption of the loaded drug through intact corneal tissues.

## 4. Conclusions

CIP-loaded NLCs and the IG formulations were successfully prepared and optimized, using gellan gum as a gelling agent. The ex vivo studies showed improved transcorneal permeability and flux when compared to the control solution. Overall, the NLC and NLC-IG prepared in this research appear to be suitable for ocular bioavailability enhancement of CIP due to the prolonged drug residence time on the ocular surface and/or sustained drug release from the delivery system. The NLC-IG formulation could decrease frequent dosing, prolong the therapeutic effect, and increase patient compliance compared to the conventional dosage form. Sterilization, in vivo ocular biodistribution and efficacy evaluation in a rabbit BE model are additional studies that are needed for the formulation to be developed into an ocular dosage form for the treatment of BE. Thus, the NLCs combined with an ion-sensitive in situ gelling agent present an efficient topical drug delivery platform.

## Figures and Tables

**Table 1:** Screening of solid and liquid lipids for Ciprofloxacin containing nanostructured lipid carriers (CIP-NLC) (Drug and lipids added in 1:10 ratio; 80 ± 2 °C; under continuous magnetic stirring at 2000 rpm for 10 mins).

Solid/Liquid-Lipid	Lipid	Solubility
Solid	Precirol^®^ ATO 5	(+)
	Compritol^®^ 888 ATO	(−)
	Dynasan^TM^ 114	(−)
	Geleol^TM^	(−)
	Gelucire^TM^ 43/01	(−)
	Dynasan^TM^ 116	(−)
	Gelucire^TM^ 50/13	(−)
	Gelucire^TM^ 44/14	(−)
	Softisan 154	(−)
Liquid	soybean oil	(−)
	Captex^®^ 355 EP	(−)
	castor oil	(−)
	sesame oil	(−)
	Maisine^®^ CC	(−)
	Miglyol^®^ 829	(−)
	Oleic acid	(+)
	Capryol 90TM	(−)
	Olive oil	(−)

(+): CIP is soluble in the melted lipid and does not precipitate on cooling; (−): CIP is either soluble in the molten lipid but precipitates on cooling or is insoluble in the lipid.

**Table 2:** Composition of optimized CIP-NLC and CIP-NLC loaded in situ gel system (CIP-NLC-IG) formulations.

Formulation Composition (%*w*/*v*)	CIP-NLC	CIP-NLC-IG
Ciprofloxacin	0.1	0.1
Precirol^®^ ATO 5	3	3
Oleic acid	1	1
Tween^®^ 80	2	2
Poloxamer 188	0.25	0.25
Glycerin	2.25	2.25
Gellan gum	-	0.2
Water	Up to 10 mL	Up to 10 mL

**Table 3:** Effect of different Tween^®^ 80 concentrations on particle size, polydispersity index and zeta potential of NLC placebos (mean ± SD, *n* = 3).

Composition (%*w*/*v*)	O-NLC-75	O-NLC-100	O-NLC-150	O-NLC-200
Precirol^®^ ATO 5	3	3	3	3
Oleic acid	1	1	1	1
Tween^®^ 80	0.75	1	1.5	2
Poloxamer 188	0.25	0.25	0.25	0.25
Glycerin	2.25	2.25	2.25	2.25
Water	Up to 10 mL	Up to 10 mL	Up to 10 mL	Up to 10 mL
PS (nm)	384.4 ± 7.9	291.6 ± 10.4	211.7 ± 4.5	142.3 ± 3.9
PDI	0.41 ± 0.06	0.42 ± 0.08	0.39 ± 0.02	0.38 ± 0.01
ZP (mV)	−28.5 ± 1.5	−29.6 ± 0.7	−31.4 ± 1.3	−27.1 ± 1.7

**Table 4:** Effect of lipids to surfactant ratio on particle size, polydispersity index and zeta potential of NLC placebos (mean ± SD, *n* = 3).

Formulation Composition (%*w*/*v*)	O-NLC-200	O-NLC
Precirol^®^ ATO 5	3	4.5
Oleic acid	1	1.5
Tween^®^ 80	2	2
Poloxamer 188	0.25	0.25
Glycerin	2.25	2.25
Water	Up to 10 mL	Up to 10 mL
PS (nm)	142.3 ± 3.9	226.0 ± 11.2
PDI	0.38 ± 0.01	0.60 ± 0.05
ZP (mV)	−27.1 ± 1.7	−27.8 ± 0.5

**Table 5:** Rheological evaluation of CIP-NLC-IG with different gellan gum concentrations (mean ± SD, *n* = 3).

Formulation	Gellan Gum (%*w*/*v*)	GT	GRT (h)	η (cP) Without STF	η (cP) With STF	Drug Content (%)
CIP-NLC-IG2	0.2	Immediate	>24	7.9 ± 0.2	55 ± 2.7	94.8 ± 2.4
CIP-NLC-IG3	0.3	Immediate	>24	25.4 ± 2.3	110 ± 3.5	83.1 ± 1.8
CIP-NLC-IG4	0.4	Immediate	>24	93.4 ± 4.6	211.9 ± 8.7	77.4 ± 2.0

**Table 6:** Mathematical model fitting of release kinetics of CIP from optimized CIP-NLC, optimized CIP-NLC-IG, and CIP-C formulations.

Model	Equation	R^2^ Value		
		CIP-C	CIP-NLC	CIP-NLC-IG2
Zero-order	*Q*_0_ − *Q = kt*	0.697	0.930	0.976
First order	*ln Q = kt*	0.797	0.984	0.995
Higuchi’s matrix	*Q*_0_ − *Q = kt*^1/2^	0.927	0.986	0.969
Korsemeyer–Peppas	*log (Q*_0_ − *Q) = n log t + log k*	0.99	0.995	0.998

Where, *Q*_0_ and *Q* is initial drug content at time *t*_0_ and drug content at time *t*, respectively; Zero-order model: % drug released vs. time; First order model: ln amount drug remaining vs. time; Higuchi model: % drug released vs. square root of time; Korsmeyer–Peppas model: *log* % drug released vs. log time.

**Table 7:** Transcorneal flux and permeability of CIP from CIP-NLC, CIP-NLC-IG and CIP-C formulation through isolated rabbit cornea (Mean ± SD, *n* = 3).

Formulation	Flux (µg/min/cm^2^)	Permeability (×10^−5^ cm/min)	Fold Enhancement with CIP-C	
			Flux	*p*
**CIP-C**	0.04 ± 0.01	2.3 ± 0.8	-	-
**CIP-NLC**	0.16 ± 0.01 ^#^	8.1 ± 0.4 ^#^	4	3.5
**CIP-NLC-IG2**	0.09 ± 0.01 ^#^	4.4 ± 0.4 ^#^	2.2	1.9

^#^ indicates statistically significant at *p* < 0.05 compared with CIP-C formulation.
